# Vegetation state changes in the course of shrub encroachment in an African savanna since about 1850 CE and their potential drivers

**DOI:** 10.1002/ece3.5955

**Published:** 2019-12-30

**Authors:** Ximena Tabares, Heike Zimmermann, Elisabeth Dietze, Gregor Ratzmann, Lukas Belz, Andrea Vieth‐Hillebrand, Lydie Dupont, Heinz Wilkes, Benjamin Mapani, Ulrike Herzschuh

**Affiliations:** ^1^ Alfred Wegener Institute Helmholtz Centre for Polar and Marine Research Potsdam Germany; ^2^ Institute of Biochemistry and Biology Potsdam University Potsdam Germany; ^3^ Institute of Biology Freie Universität Berlin Berlin Germany; ^4^ Institute for Chemistry and Biology of the Marine Environment Carl von Ossietzky University of Oldenburg Oldenburg Germany; ^5^ GFZ German Research Centre for Geosciences Helmholtz Centre Potsdam Potsdam Germany; ^6^ MARUM – Centre for Marine Environmental Sciences University of Bremen Bremen Germany; ^7^ Department of Geology University of Namibia Windhoek Namibia; ^8^ Institute of Environmental Science and Geography Potsdam University Potsdam Germany

**Keywords:** climate change, fossil pollen, land‐use change, savanna ecology, sedimentary ancient DNA, state and transition, tree–grass interactions

## Abstract

1. Shrub encroachment has far‐reaching ecological and economic consequences in many ecosystems worldwide. Yet, compositional changes associated with shrub encroachment are often overlooked despite having important effects on ecosystem functioning.
2. We document the compositional change and potential drivers for a northern Namibian *Combretum* woodland transitioning into a *Terminalia* shrubland. We use a multiproxy record (pollen, sedimentary ancient DNA, biomarkers, compound‐specific carbon (δ^13^C) and deuterium (δD) isotopes, bulk carbon isotopes (δ^13^Corg), grain size, geochemical properties) from Lake Otjikoto at high taxonomical and temporal resolution.
3. We provide evidence that state changes in semiarid environments may occur on a scale of one century and that transitions between stable states can span around 80 years and are characterized by a unique vegetation composition. We demonstrate that the current grass/woody ratio is exceptional for the last 170 years, as supported by *n*‐alkane distributions and the δ^13^C and δ^13^Corg records. Comparing vegetation records to environmental proxy data and census data, we infer a complex network of global and local drivers of vegetation change. While our δD record suggests physiological adaptations of woody species to higher atmospheric *p*CO_2_ concentration and drought, our vegetation records reflect the impact of broad‐scale logging for the mining industry, and the macrocharcoal record suggests a decrease in fire activity associated with the intensification of farming. Impact of selective grazing is reflected by changes in abundance and taxonomical composition of grasses and by an increase of nonpalatable and trampling‐resistant taxa. In addition, grain‐size and spore records suggest changes in the erodibility of soils because of reduced grass cover.
4. *Synthesis.* We conclude that transitions to an encroached savanna state are supported by gradual environmental changes induced by management strategies, which affected the resilience of savanna ecosystems. In addition, feedback mechanisms that reflect the interplay between management legacies and climate change maintain the encroached state.

## Graphical abstract

We present the first multidecadal time series with high taxonomic and temporal resolution, which includes *sed*aDNA, and that enables the tracking of vegetation and environmental change in a southern African savanna. According to our results, shrub encroachment occurs on a scale of one century and with a phase of transition characterized by a steady turnover of taxa, some of which are restricted to this phase. We discuss further the feedback mechanisms that stabilize the encroached state, as well as the triggers of vegetation change.

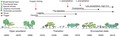

## 1 INTRODUCTION

Shrub encroachment is affecting savannas worldwide (Saha, Scanlon, & D’Odorico, 2015; Stevens, Lehmann, Murphy, & Durigan, 2017; Tian, Brandt, Liu, Rasmussen, & Fensholt, 2017). This process is assumed to indicate a savanna state change from open woodlands and grasslands, characterized by dominance of C_4_ grasses, to a bush‐thickened savanna (Joubert, Rothauge, & Smit, 2008; Meyer, Wiegand, Ward, & Moustakas, 2007). Southern Africa is a hotspot of studies reporting shrub encroachment (O’Connor, Puttick, & Hoffman, 2014; Roques, O’Connor, & Watkinson, 2001; Wigley, Bond, & Hoffman, 2009). This is not only because shrub encroachment is a widespread phenomenon, but also because the area is traditionally used as grazing grounds and encroachment reduces the land‐carrying capacity, threatening local economies (De Klerk, 2004; Moyo, O’Keefe, & Sill, 1993).

Shrub encroachment has been described as an alternative stable state occurring several times during the last two millennia in African savannas (Gil‐Romera, Lamb, Turton, Sevilla‐Callejo, & Umer, 2010). There is also some indication that African savannas are characterized by grassland/woodland phases occurring with a periodicity of 250–600 years (Gillson, 2004; Gil‐Romera et al., 2010). Such woodland phases were not always led by the spread of encroaching species (Gil‐Romera et al., 2010; Scott, Cooremans, de Wet, & Vogel, 1991), which suggests that a mere increase in woody cover, as reported by several studies since the 20th century (Hoffman, Rohde, & Gillson, 2019; van Rooyen, le Roux, van der Merwe, van Rooyen, & Geldenhuys, 2018; Wiegand, Ward, & Saltz, 2005), does not necessarily mean shrub encroachment. Accordingly, identification of encroached states should be related to the increasing cover of encroacher species and the suppression of perennial grasses (Gil‐Romera et al., 2010). However, the low taxonomic resolution of pollen records, particularly regarding Poaceae, constrains the identification of these phases on a long timescale.

It is assumed that resilience of savanna ecosystems is scale dependent. For example, savanna seems to be resilient to changes at a centennial timescale, as the vegetation fluctuates between two stable states of grassland and woodland (Gillson, 2004; Gil‐Romera et al., 2010). Conversely, savanna seems to be less resilient at a decadal scale, as encroachment may occur within a century and without regression to a grassy state even at a patch scale (Rohde & Hoffman, 2012). Similarly, African landscapes are predicted to shift to woodland states, with abrupt transitions at the local scale, but smoother at the continental scale (Higgins & Scheiter, 2012). Transitions have been described as the unstable equilibrium between the “basins of attraction” of stable states (Holling, 1973; Scheffer, Carpenter, Foley, Folke, & Walker, 2001), but surprisingly little is known about the duration of such transitions (Higgins & Scheiter, 2012; Joubert et al., 2008) or the compositional changes they entail (Joubert et al., 2008; Liao, Clark, & DeGloria, 2018).

Different opinions exist as to the causes of shrub encroachment in savannas (Devine, McDonald, Quaife, & Maclean, 2017; Venter, Cramer, & Hawkins, 2018; D Ward, 2005). Among others, changes in precipitation, atmospheric *p*CO_2_, vegetation fires, and land use are considered to be triggers and/or drivers (Aleman, Blarquez, & Staver, 2016; Berry & Kulmatiski, 2017; Buitenwerf, Bond, Stevens, & Trollope, 2012; Case & Staver, 2017), which likely interplay at different timescales. For example, while climate shifts are considered the main driver of changes in the grass/woody vegetation ratio on a long timescale (Gil‐Romera et al., 2010; Scott et al., 1991), changes in grazing pressure and fire, besides stochastic variations in rainfall, are thought to drive decadal‐scale variability (Gillson, 2004; van Rooyen et al., 2018). Interestingly, different studies have shown that precipitation, fire, and herbivory may have different effects on encroacher species, and thus a different effect on savanna stability (Joubert et al., 2008; Joubert, Smit, & Hoffman, 2012, 2013). Hence, disentangling triggers that induce changes of state, as well as understanding stabilizing feedbacks, requires long time series with adequate taxonomic resolution.

In addition, gradual change in environmental conditions is assumed to have little effect on the feedbacks that support a stable state but still alters its resilience (Scheffer et al., 2001). For example, management legacies are assumed to affect the properties of temperate woodland responses to current environmental change up to 200 years after disturbance (Perring et al., 2018). Similarly, shrub encroachment of Namibian savannas has been related to management strategies during colonial administration (Moyo et al., 1993; Rohde & Hoffman, 2012; Verlinden & Kruger, 2007). However, it remains unclear to what extent management may have affected species composition and thus savanna stability in Namibia.

Lake sediments represent unique environmental archives. Traditionally, they are mostly used to resolve millennial‐scale environmental changes in savannas (Metwally, Scott, Neumann, Bamford, & Oberhänsli, 2014; Quick et al., 2018). Vegetation reconstruction using pollen deposited in lakes has been demonstrated to be a powerful method to track structural and compositional changes in savannas despite the low pollen taxonomic resolution (Miller & Gosling, 2014; Neumann, Scott, Bousman, & van As, 2010). Recently, sedimentary ancient DNA (*sed*aDNA) using the g‐h universal primers (Taberlet et al., 2007) was established as a further proxy. This technique enables the reconstruction of tropical plant diversity at high taxonomic resolution (Boessenkool et al., 2014; Bremond et al., 2017). Further, fire dynamics and its impact on savanna vegetation can be reconstructed from sedimentary charcoal (Colombaroli, van der Plas, Rucina, & Verschuren, 2018; Gillson & Ekblom, 2009), while carbon (δ^13^C) and deuterium (δD) isotope compositions of plant‐wax *n*‐alkanes can be used to reconstruct vegetation structure and hydrological variability (Garcin et al., 2018; Miller et al., 2019; Walther & Neumann, 2011). In addition, grain‐size analyses of lake sediments are useful to reconstruct sediment mobility in savanna landscapes and to identify soil erosion (Walther & Neumann, 2011). However, high‐resolution multiproxy approaches that include the use of *seda*DNA, and that track vegetation and environmental changes in semiarid savannas at a decadal scale, are still missing.

**Figure 1:**
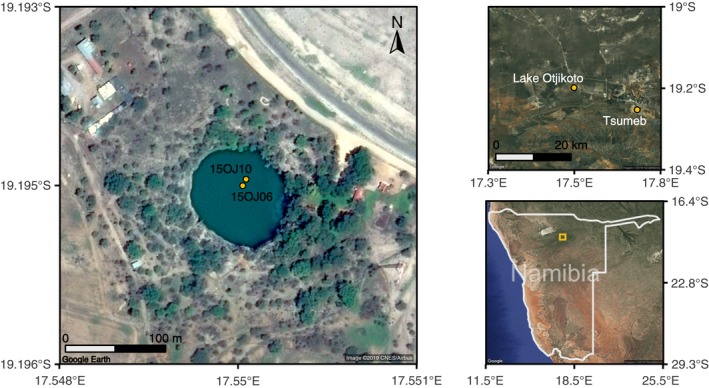
Map of the study site in Namibia

Lakes are rare in southern Africa. Lake Otjikoto, a sinkhole lake located in the Karstveld area in northern Namibia (Figure 1), represents a unique environmental archive (Scott et al., 1991). Applying a multiproxy approach to lake sediments from Otjikoto including the analyses of pollen, *sed*aDNA, biomarkers, geochemical proxies, grain size, and macrocharcoal analyses, this study provides the first decadal‐scale record of the turnover of open savanna woodlands to shrublands from northern Namibia in the period between about 1,850 and the present day. This enables the discussion of savanna state changes and, in combination with climate and land‐use data, the investigation of potential triggers and drivers and related feedbacks of the vegetation.

## 2 MATERIALS AND METHODS

### 2.1 Site

The lake basin is formed from a groundwater cave, which developed from soluble dolomite of the Tsumeb subgroup of the Damara sequence (Kamona & Günzel, 2007). Water depth is ~71 m; diameter is ~102 m. Water pH measurements vary between 7.0 and 8.7 (Marchant, 1980). The potential natural vegetation in the vicinity of the lake comprises *Terminalia prunioides‐Combretum apiculatum* woodlands (Mendelsohn, El Obeid, & Roberts, 2000). Accordingly, a low grass layer and dominance of trees characterize this vegetation type, which is common on shallow sandy–loamy soils. *Terminalia prunioides, C. apiculatum, C. imberbe, Commiphora glandulosa*, *Vachellia reficiens, V. hebeclada,* and *Senegalia mellifera* are the most common taxa. The shrub layer comprises *Grewia* sp., *Gymnosporia senegalensis*, *Dichrostachys cinerea,* and *Croton gratissimus.* However, a dense shrubland characterizes the current local vegetation, which is mainly composed of *T. prunioides, S. mellifera, D. cinerea, Ziziphus mucronata,* and *Grewia* sp. Shore vegetation is sparse, with *Sclerocarya birrea* and *Ficus* growing on the steep rock walls surrounding the lake. Crop fields are close to the lake. Mean annual precipitation is 456 mm, falling mainly in the summer months (November–March); mean monthly temperature in summer is 27°C and in winter 15°C (Tsumeb climate station; Harris, Jones, Osborn, & Lister, 2014). Cattle farming is the dominant land use.

### 2.2 Materials

Two cores—15OJ10 (length 31 cm, Ø = 6 cm) and 15OJ06 (length 30 cm, Ø = 6 cm)—were collected in March 2015 using UWITEC coring equipment at a water depth of 50 m (19.19467°S, 17.54980°E). The core extraction was carried out with a pulley and without motor support. Divers from Dantica diving club Windhoek assisted with the core extraction from the lake bed. Core 15OJ10 was sampled at a 0.5 cm resolution in the field. We obtained 61 samples for pollen analysis; 30 subsamples were used for sedimentological, geochemical, *n*‐alkane, and isotope analyses. Core 15OJ06 was sampled at a 1.5 cm resolution at the Alfred Wegener Institute, Helmholtz Centre for Polar and Marine Research. We obtained 21 samples for *sed*aDNA and charcoal analysis. All samples were stored at 4°C.

### 2.3 Dating

Sixteen subsamples from core 15OJ10 and 21 from core 15OJ06 were analyzed for ^210^Pb, ^226^Ra, and ^137^Cs by direct gamma assay at the Liverpool University Environmental Radioactivity Laboratory. Measures were obtained using an Ortec HPGe‐GWL well‐type coaxial low background intrinsic germanium detector (Appleby et al., 1986). ^210^Pb dates were calculated using the CRS dating model (Appleby & Oldfield, 1978). Corrections were made using the ^137^Cs date 1964 as a reference point (Appleby, 2001).

### 2.4 Pollen and palynomorph analysis

Pollen extraction was performed using standard procedures (10% HCl, KOH, 40% HF—including 4 hr boiling—and acetolysis) (Faegri & Iversen, 1989; Moore, Webb, & Collison, 1991). We added one tablet of *Lycopodium* spores (batch 1,031) to each sample to calculate pollen concentrations (Stockmarr, 1971).

At least 400 pollen grains were counted per sample using a microscope of x400 magnification. Critical identification was made by x1,000 magnification based on standard literature (Bonnefille & Riollet, 1980; Gosling, Miller, & Livingstone, 2013; Leźine, 2005; Schüler & Hemp, 2016; Scott, 1982), taxonomical studies (Banks & Lewis, 2018; Poston & Nowicke, 1993), and online pollen catalogues (Bremond, 2011). We differentiate the *Acacia* type into *Vachellia* and *Senegalia* by means of columella identification (Kyalangalilwa, Boatwright, Daru, Maurin, & van der Bank, 2013). Spores’ identification follows Gelorini, Verbeken, Geel, Cocquyt, and Verschuren (2011). Pollen percentages were calculated based on the total terrestrial pollen sum per sample. Shore taxa (*Ficus,* Cyperaceae) and aquatic taxa were not included in the pollen sum.

### 2.5 Sedimentary ancient DNA analysis

The sampling of core 15OJ06 for *sed*aDNA was carried out at +10°C in a climate chamber that was previously cleaned (DNA‐ExitusPlus^TM^—AppliChem, Germany; deionized water and ethanol 100%) to prevent contamination. Extraction and polymerase chain reaction (PCR) followed the same procedures as described in Zimmermann et al. (2017). For each sample (*n* = 21) and extraction negative control (*n* = 3), we performed six PCR replicates to ensure the accurate detection of taxa (Ficetola et al., 2015). For each PCR, we used two negative controls and different primer tag combinations leading to a total of 126 PCR products and 30 negative controls. All PCR products were purified using the MinElute PCR Purification Kit (Qiagen, Hilden, Germany) according to the manufacturer's instructions. DNA concentrations were measured with the dsDNA BR Assay Kit (Life Technologies, USA) using 1 µl of the purified amplifications with the Qubit 2.0 fluorometer (Invitrogen, Carlsbad, CA, USA). Purified samples were pooled in equal concentrations and sent to the Fasteris SA sequencing service (Geneva, Switzerland) for library preparation following the MetaFast protocol. Sequencing was realized on an Illumina HiSeq 2,500 platform (2 × 125 bp) (Illumina Inc., San Diego, CA, USA) using 1/10 of a flow cell lane. Obtained data were processed and assigned their taxonomic name using the software package OBITools (Boyer et al., 2016) following the descriptions in Zimmermann et al. (2017). Sequences selected for further analyses needed at least 95% best identity with respect to an entry in the reference database and at least 10 sequence counts in at least two samples in the dataset. Taxa percentages were calculated based on the sum of sequence counts per sample. After sequencing, two replicate batches were excluded from the analysis because they lacked sequencing depth, probably due to errors in the allocation of tags.

### 2.6 Biomarker analysis

For the analysis of *n*‐alkanes, 30 freeze‐dried sediment samples were extracted using a dichloromethane containing 1% methanol. Further procedures used for the analysis of *n*‐alkanes follow those described by Vogts, Moossen, Rommerskirchen, and Rullkötter (2009) and Badewien, Vogts, Dupont, and Rullkötter (2015).

The carbon (δ^13^C) and deuterium (δD) isotopic compositions of saturated hydrocarbons were measured using gas chromatography isotope ratio mass spectrometry consisting of a GC‐Unit (7890N; Agilent Technology, USA) connected to GC‐Isolink and coupled to a Delta V Plus mass spectrometer (Thermo Fisher Scientific, Germany). We used a combustion interface for δ^13^C, and a pyrolysis furnace for δD (Supplement 1.A). Isotope measurements were controlled based on *n*‐alkane standards with known isotopic composition (Campro Scientific, Germany, and Arndt Schimmelmann, Indiana University, USA).

Isotope measurements of bulk organic carbon (δ^13^Corg) were made on decarbonized samples using an elemental analyser (1,108 from Carlo Erba) coupled to a MAT 252 isotope ratio mass spectrometer from Thermo Fisher Scientific (Supplement 1B). All subsamples were analyzed in duplicate.

### 2.7 Charcoal analysis

Charcoal was extracted from 1 cm^3^ subsamples following Mooney and Tinner (2011). Samples were bleached (24 hr) and washed through a sieve (125 µm) to separate macrocharcoal particles. All charcoal particles (>125 µm) were counted for each sample using a binocular microscope at ×15 magnification and a Bogorov counting chamber.

### 2.8 Geochemical analysis

Total carbon (TC) and total nitrogen (TN) contents were analyzed for 30 freeze‐dried subsamples (core 15OJ10) using an Elementar vario EL III (CNS) analyser. An Elementar vario MAX C was used to quantify total organic carbon (TOC). Total inorganic carbon (TIC) was calculated by deducting TOC from TC values.

### 2.9 Grain‐size and end‐member modeling analysis (EMMA)

Samples were pretreated with CH_3_COOH (10% for 24 hr) to remove carbonates, and with H_2_O_2_ (initial 0.3%, with addition of 10 ml (35%) every second day for up to six weeks) to remove the organic sediment fraction. Grain‐size analysis was performed using a Coulter LS 200 Laser Diffraction Particle 30 Analyser.

Robust grain‐size end‐members (EMs) have been modeled from all grain‐size distributions using the EMMA algorithm of Dietze et al. (2012). We used the compact protocol in the R package EMMAgeo (Dietze & Dietze, in press). Definition of the robust EMs considered the main mode classes that were modeled most frequently (see kernel density estimate in Figure S2a), when the number of end‐members and weight transformation limit was varied between a minimum and maximum value (after Dietze et al. (2012) and Dietze & Dietze, in press). EM loadings (Figure S2b) represent scaled grain‐size classwise EM contributions, and scores (Figure S2c) represent the contribution of EMs in the samples.

## 3 RESULTS

### 3.1 Chronology

The core 15OJ10 covers the period from 1886 to 2015. The results suggest a relatively steady accumulation since the late 1980s with a mean sedimentation rate of 0.34 cm/year. Sedimentation rates in the 1930s were around 0.21 cm/year, increasing to around 0.31 cm/year by the early 1980s (Figure S3a). Low ^210^Pb concentrations in the pre‐1930 samples made accurate dating problematic. However, ^210^Pb dates suggest even lower sedimentation rates in the late 19th and early 20th century with a mean value of 0.15 cm/year. We applied linear interpolation to obtain the chronology of segments between dated samples.

The core 15OJ06 covers the period from 1849 to 2015. Since the ^210^Pb concentrations reach the limit of detection at a depth of 23.25 cm (dated to 1907), we calculated ages for samples below 23 cm by linear extrapolation. The sedimentation rate since the early 1980s has a mean accumulation value of 0.30 cm/year (Figure S3b). The results show lower sedimentation rates increasing from 0.14 cm/year during the first few decades of the 20th century to 0.20 cm/year during the period from 1940 to the 1970s.

### 3.2 Pollen and nonpollen palynomorph records

**Figure 2:**
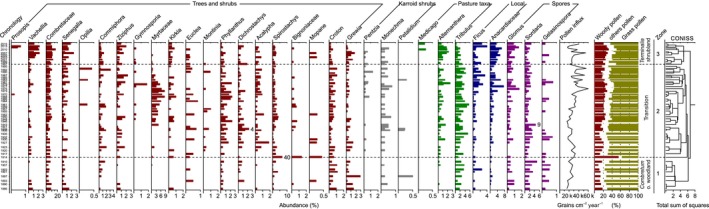
Percentage abundance diagram of selected pollen and spore taxa. Arrangement of taxa follows their dominance in the pollen zones (separated by dotted lines). Taxa are displayed in subgroups: trees and shrubs (brown), Karroid shrubs (gray), taxa associated with pastures (green), local shore taxa (blue), and spores (purple). Pollen influx, as well as mean abundance percentages of woody, grass, and other pollen taxa, is displayed. Truncated values are indicated on the bars

We identified 116 pollen and spore taxa (Table S4). The most dominant taxa are Poaceae, Anacardiaceae, and Combretaceae. The pollen diagram captures a signal of grasses decreasing up the core, with values dropping below 60% in the 1970s and from the turn of this century, while the pollen signal of woody vegetation increases (Figure 2).

Based on a visual inspection of all proxies of both cores and guided by the results of the cluster analysis of the pollen record, we defined three zones. The taxa association in zone 1 (1886–1910) comprises Combretaceae, *Dichrostachys, Croton*, *Grewia, Euclea*, *Senegalia,* and *Petalidium*. Bignoniaceae, *Spirostachys,* and *Colophospermum mopane* yield high percentages at the border to zone 2. The pollen signals of *Dichrostachys* and *Ziziphus* are high in zone 2 (1914–1997), while *Grewia* is lower compared with zone 1. Furthermore, zone 2 is characterized by high abundance of *Myrtaceae*, as well of *Alternanthera*, *Tribulus*, *Pentzia*, *Monechma*, *Opilia, Montinia*, *Gymnosporia,* and Euphorbiaceae taxa. Zone 3 (1998–2015) is characterized by high values in *Vachellia, Senegalia*, *Grewia,* and *Ziziphus*. This goes together with an increased signal of *Alternanthera* and low values for *Euclea*. The signal of *Prosopis* increases toward the top.

NPP results show a gradual decrease of *Gelasinospora* and *Sordaria* toward the top of the core. *Glomus* reaches highest values in zone 3.

### 3.3 Sedimentary ancient DNA records

**Figure 3:**
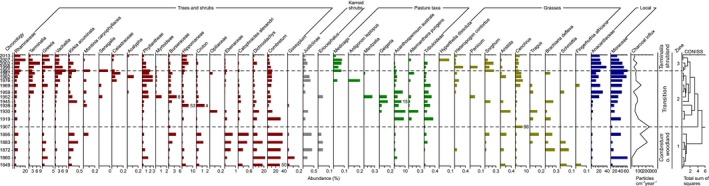
Diagram of selected *sed*aDNA taxa. Arrangement of taxa follows their dominance in the *sed*aDNA zones (separated by dotted lines). Taxa are displayed in subgroups: trees and shrubs (brown), Karroid shrubs (gray), taxa associated with pastures (green), grasses (yellow), and local shore taxa (blue). Macrocharcoal influx is also displayed

We obtained 4,211,287 sequence counts (sqc) from Illumina sequencing for 21 samples with four replicates. After bioinformatic filtering, 1,840,462 sqc were assigned to 177 taxa (Table S5). Of these, 37 could be identified to family level (905,299 sqc), 58 to genus level (323,335 sqc), and 64 to species level (239,924 sqc). Most dominant taxa are *Moraceae, Combretum,* and Anacardiaceae. Overall, the *sed*aDNA record reflects a turnover from taxa characteristic of open savanna woodlands in zone 1 to shrubland taxa in zone 3 (Figure 3).

Zone 1 (1849–1907) is characterized by dominance of *Dichrostachys, Combretum,* Ebenaceae, and *Catophractes alexandri* and the occurrence of *Gossypium*. A mixed grass layer consisting of perennial grasses such as *Fingerhutia africana*, *Schmidtia,* and *Cenchrus* and annual grasses such as *Brachiaria deflexa, Aristida,* and *Tragus* characterizes zones 1 and 2, as well as the occurrence of Karroid shrubs. Zone 2 (1919–1991) is distinguished by a high abundance of taxa associated with pastures such as *Antigonon leptopus, Mentzelia*, Tribuloideae, *Alternanthera pungens, Geigeria,* and *Acanthospermum,* as well as of shrub taxa from the Euphorbiaceae, Celastraceae, and Opiliaceae families. In zone 2, the signals of Myrtoideae are high. Zone 3 (1996–2015) is characterized by high signals of *Terminalia, Vachellia,* and *Senegalia,* as well as *Grewia,* Rhamnaceae, and *Medicago*. The perennial grasses *Hyperthelia dissoluta*, *Panicum,* and *Heteropogon contortus* characterize zone 3. Furthermore, the relative abundance of Moraceae increases in the upper part of the core.

### 3.4 Biogeochemical and macrocharcoal analysis

**Figure 4:**
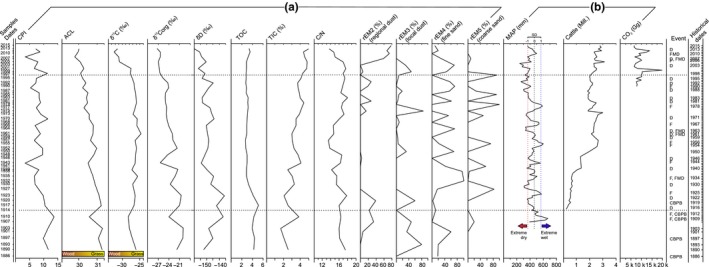
(A) Biogeochemical and sedimentological proxies from core 15OJ10: Carbon preference indices (CPI_25‐35_); average chain length of *n*‐alkanes with an odd number of carbon atoms (ACL_27‐33_); weighted mean of carbon isotopic compositions of *n*‐alkanes (δ^13^C_WMA27‐33_); carbon isotopic composition of organic matter (δ^13^Corg); weighted mean of deuterium isotopic compositions of *n*‐alkanes (δD_WMA27‐33_); total organic carbon (TOC) content; total inorganic carbon (TIC) content; total organic carbon to nitrogen ratio (C/N); and scores from robust end‐member loadings (rEM). Dotted lines indicate pollen zones. (B) Historical records: Mean annual precipitation (3‐year running mean—MAP) was calculated from climate data (meteorological station Tsumeb), vertical black dotted line corresponds to the long‐term MAP, and red dotted lines correspond to the standard deviation (*SD*); livestock numbers Namibia (data 1915–1959, 1961, 2015; Lange et al., 1998; FAO, 2018b); CO_2_ emissions Namibia (FAO, 2018a); documented events (D: drought; F: flood; FMD: foot‐and‐mouth disease; CBPP: contagious bovine pleuropneumonia (Directorate of Planning, 2011, 2005; Nicholson & Selato, 2000; Schneider, 2012; Sweet, 1998))

Concentration of organic and inorganic carbon (TOC, TIC) is highest in zones 1 (5.1%) and 3 (4.9%), respectively (Figure 4). The average chain length (ACL; Poynter, 1989) values for *n*‐alkanes with an odd number of carbon atoms (C_27_‐C_33_) range from 31.1 to 30.3 and increase with depth. The carbon preference indices (CPI_25‐35_; Marzi, Torkelson, & Olson, 1993) range from 13.6 to 3.4 with an average of 8.4. The C/N ratios range from 18.05 to 13.4 with an average of 16.04. The carbon isotopic compositions of organic matter (δ^13^Corg) range from −19.9 to −27.3 with an average of −24.0‰ and with the more ^13^C‐depleted values in zone 3. Similarly, the weighted mean δ^13^C values of *n*‐alkanes with an odd number of carbon atoms (δ^13^C_WMA27‐33_) range from −24.6‰ to −32.3‰ and have the more ^13^C‐depleted values in zone 3. We also find the more depleted weighted mean δD values (δD_WMA27‐33;_ range: −135.7‰ to −159.6‰) in this zone. In addition, macrocharcoal flux is highest in zones 1 and 2 and decreases upwards in the core (Figure 3).

### 3.5 Grain‐size end‐members

We obtained five rEM (robust grain‐size end‐members) after calculating similarly–likely EM models (Figure S2). The mean rEMs explain 77% of the mean total variance of the original grain‐size dataset. The rEM1 (23% explained variance) has a primary mode between 0.2 and 14.5 µm with a maximum at 1.8 µm, covering the clay and very fine silt fraction. The rEM2 (13% explained variance) has a broad primary mode between 3 and 66 µm covering the whole silt fraction, with a maximum at 18.7µm, which corresponds to medium silt. The rEM3 (14% explained variance) has a mean mode between 16 and 144µm, and a maximum at 51µm (medium silt to fine sand fraction). The rEM4 (23% explained variance) has a very robust mode in the very fine to fine sand between 51 and 310 µm, with a maximum at 127 µm. The coarsest component rEM5 (26% explained variance) has a robust mode in the fine to medium sand between 86 and 454 µm, and a maximum at 211 µm.

## 4 DISCUSSION

### 4.1 Source of the lake sediments

We consider terrestrial plants to be a major source of organic matter in the sediments of Lake Otjikoto due to their high CPI_25‐35_ and C/N values (Meyers, 1997). Although higher TOC values (>4%) below 25 cm depth, and the oscillating variation of δ^13^Corg content below 18 cm, suggest a bacterial and algal contribution to the sediments (cf. Kristen et al.[, 2007]), corresponding C/N values (>16) indicate a higher proportion of allochthonous input relative to the autochthonous component of the organic matter (Meyers, 1997).

### 4.2 Savanna state change: from open woodland to dense shrubland

#### 4.2.1 Multidecadal shift in the grass/woody ratio

Our multiproxy approach indicates a shift in the dominance among grasses and woody vegetation. In particular, our results show an increase in woody vegetation since about 1915 in the Tsumeb region with a particularly strong increase after about 1998. These results validate vegetation models that suggest an increase in woody cover at low rates since 1,850 in Africa, and an accelerated rate between 1990 and 2010 (Higgins & Scheiter, 2012). Our findings also support results from remote‐sensing analyses of sub‐Saharan savannas, which have observed an increase in woody vegetation cover over the last three decades (Mitchard & Flintrop, 2013; Venter et al., 2018).

The ratio of Poaceae/woody pollen (Figure 2) indicates that the current grass/woody ratio is exceptional for the last 170 years and is similar to the ratio found by Scott et al. (1991) in a previous palynological study from Lake Otjikoto. Scott et al. (1991) identified two earlier woodland phases with woody pollen abundances up to 33% and Poaceae pollen below 60% (Figure S6). In our study, we estimate grass pollen abundances to decrease to below 60% in the 1970s and in the 21st century, while woody pollen percentages increase up to 32% suggesting a woodland phase in zone 3. These results are consistent with a modern pollen‐vegetation study conducted in the vicinity of the lake, which found a mean woody pollen abundance of 33% by a mean woody cover of 74% at the study site (Tabares, Mapani, Blaum, & Herzschuh, 2018).

Biogeochemical results also support our pollen findings. The relatively constant δ^13^C_WMA27‐33_ values throughout zones 1 and 2 indicate mixed C_3_/C_4_ vegetation with the C_4_ proportion generally above 50% (Badewien, Vogts, & Rullkötter, 2015). Only in the very recent past (zone 3) does δ^13^C_WMA27‐33_ shift toward more ^13^C‐depleted values providing evidence for an increase of the C_3_ proportion up to around 80%, which is also supported by δ^13^Corg values around −27‰ (Meyers, 1997). It has been reported that southern African savanna trees show significant variations in their ACL values, which, for example, are higher for *C. apiculatum* and *C. mopane* than for *T. prunioides* and *Ziziphus* spp. (Kristen et al., 2010; Vogts et al., 2009; for Australian trees, see Hoffmann, Kahmen, Cernusak, Arndt, & Sachse, 2013). Assuming a significant contribution of trees to the sedimentary *n*‐alkanes, the overall shift of ACL toward lower values throughout the profile is thus in agreement with the vegetation shifts inferred from pollen analyses and *seda*DNA as discussed in detail below.

In addition, grain‐size distribution lets us infer changes in landscape openness via the type of sediment transport reflected in the grain‐size rEM (Dietze et al., 2014). The two types of sand deposits represented by rEM4 and rEM5 indicate short‐term, high energetic sediment input from run‐off during rain events or sand storms in an open landscape with reduced grass cover (Dietze et al., 2014; Walther & Neumann, 2011). In particular, within the error of the age model, rEM5 can be associated with the main flood events documented in the region from the 1920s until 2000 (Figure 4). We assume that an increased woody cover (as observed from the 21st century) favors the development of soil crusts on bare patches by constraining trampling (Thomas & Dougill, 2007). Although soil crusts reduce infiltration and promote run‐off and erosion, they also increase soil roughness limiting the detachment of soil particles (Bullard, Ockelford, Strong, & Aubault, 2018; Rodríguez‐Caballero, Cantón, Chamizo, Afana, & Solé‐Benet, 2012; Valentin, 1993). Accordingly, soil crusts may retain the coarser sand fractions, which would explain the low percentages of rEM4 and rEM5 in zone 3. However, prolonged drought (as observed in the 1990s), followed by heavy rains, can damage the crust surfaces (Kidron, Ying, Starinsky, & Herzberg, 2017; Thomas & Dougill, 2007), causing the loss of fine sand‐sized particles (100–200 μm), which require less energy to be detached compared to coarser and finer‐sized particles (Bullard et al., 2018; Salles, Poesen, & Govers, 2000). This would explain why only fine sand (rEM4) mobilized during La Niña flood in 2006 (Figure 4).

An increased woody cover would also retain the local dust sedimentation (rEM3), by reducing the near‐surface wind energies. Hence, the associated importance of more remote dust (rEM2) from several hundred kilometers away within the detrital sediment fraction is seen during the last decade.

#### 4.2.2 Compositional turnover in the course of shrub encroachment

Our pollen and *sed*aDNA results indicate a savanna state change from open *Combretum* woodland to *Terminalia* shrubland during the 20th century at local (i.e., the vicinity of Otjikoto) and probably regional (i.e., the wider Tsumeb area) scales. These results are the first time series with sufficient taxonomic resolution to show compositional change in the course of shrub encroachment in a semiarid savanna at a multidecadal scale. They confirm theoretical assumptions (Joubert et al., 2008; Li, 2002) which were based on compositional observations of only a few decades or on the analyses of spatial gradients (van Rooyen et al., 2018; Strohbach, 2001).

According to our proxies, an open *Combretum* woodland dominated the landscape around Lake Otjikoto until the turn of the 19th to 20th century (i.e., zone 1 in our record). A high grass cover and the dominance of *Combretum* species in the tree layer are characteristic of this vegetation type (Mendelsohn et al., 2000), which is represented by high Poaceae pollen values and up to 50% *Combretum* in the *sed*aDNA records. Our results also show that *Grewia, D. cinerea*, *Croton, Kirkia acuminata*, *C. alexandri* (Bignoniaceae), and Ebenaceae (likely *Euclea *sp.) were common taxa in the tree and shrub layer of this vegetation type. This aligns well with modern observations of this vegetation type preserved on flat areas between dolomite hills (Giess, 1971), particularly on moderately deep soils (Mendelsohn et al., 2000). In addition, our results reflect botanical observations from the early 20th century of the existence of woodlands and lime steppes in the Tsumeb region (Dinter, 1918). A high pollen signal of *Petalidium* (likely *P. eurychlamys*) in the late 19th century and the *sed*aDNA record of *Gossypium* reflect early observations regarding both taxa as local indicators of open broad‐leaved savanna vegetation (Dinter, 1922; Mildbraed, 1941). We assume that open *Combretum* woodlands represent the potential natural vegetation of the region and a stable savanna state. These results confirm a previous low‐resolution pollen record from Lake Otjikoto (Scott et al., 1991) that revealed the presence of open Combretaceae‐*Spirostachys* woodlands in the region during the late Holocene.

Our results allow us to infer a transition phase that ranges from 1920 to about 1997 (zone 2 in our record) and provide empirical evidence to previous studies, which assume a transition period of several decades between alternative stable grassland/woodland states (Higgins & Scheiter, 2012; Joubert et al., 2008).

The transition is marked by a high turnover in the vegetation proxy composition. While Poaceae are still abundant during this period, some shrub taxa are also common (*Ziziphus* (Rhamnaceae)*, Grewia, Phyllanthus/*Phyllantheae) particularly during the second half of the 20th century. Furthermore, this transition phase is characterized by unique taxa, particularly those associated with disturbance (e.g., common pasture taxa such as *Antigonon* and *Mentzelia* (López‐Olmedo, Meave, & Pérez‐García, 2007), and nonpalatable weeds such as *A. pungens, Tribulus/*Tribuloideae, and *Geigeria* (Tabares et al., 2018)). This transition is also distinguished by the propagation of dwarf shrubs (*Monechma/*Justiciinae, *Pentzia*), annual grasses (*Tragus, B. deflexa, Aristida*), and the increase of shrub taxa (*Croton, Euphorbia, Gymnosporia* (Celastraceae), *Montinia caryophyllacea*, *Opilia*). We assume that our inferred transition phase corresponds to the unstable state described by Joubert et al. (2008), which is distinguished by the above‐mentioned characteristics and by the spread of encroacher seedlings (e.g., *S. mellifera*) in a mixed grass sward. The increase of *Senegalia* pollen in zone 3 may therefore indicate the maturity of *S. mellifera* trees established in zone 2.

The vegetation at the local and regional scale around Lake Otjikoto after 1997 (zone 3 in our record) can be characterized as a *T. prunioides* shrubland (*T. prunioides* association in Hüttich et al., 2009; Mendelsohn et al., 2000; *Acacia‐T. prunioides* in Strohbach, 2014). This represents a stable encroached savanna state (Joubert et al., 2008) and is supported by the finding that the compositional change within zone 3 is rather small compared to zone 2. The vegetation is characterized by high abundances of *Vachellia, Senegalia,* and *Terminalia* and the decline of *Combretum* (Figure 3) in our sedimentary vegetation proxy records, which likely indicates the spread of local encroacher species such as *V. reficiens, S. mellifera,* and *T. prunioides* in the region (De Klerk, 2004; Strohbach, 2001). The transition toward *T. prunioides* vegetation type is also reflected by the changes in the shrub layer: The abundances of *Grewia* and *Ziziphus* (Rhamnaceae) increase, while *C. alexandri* (Bignoniaceae) and *Euclea* (Ebenaceae) decrease. Furthermore, a state change toward encroached savanna is indicated by the decline in Poaceae pollen signals suggesting a reduction in grass cover, which is characteristic of *T. prunioides* shrubland (Strohbach, 2014). Interestingly, the increase of Fabaceae (e.g., *Vachellia, Senegalia* in pollen and *sed*aDNA values) in zone 3 corresponds with more depleted δ^13^C values of organic matter. This matches modern studies along spatial gradients that likewise found depleted δ^13^C values in Fabaceae C_3_ plant matter (Badewien, Vogts, & Rullkötter, 2015; Vogts et al., 2009).

We hypothesize that the current *Terminalia* encroached state may have occurred earlier in the region, as suggested by the compositional changes reflected in an early pollen record of Lake Otjikoto (Scott et al., 1991). In particular, the early woodland phase (zone O1), which is dominated by Combretaceae, may correspond to a phase of *Terminalia* encroachment, since the abundance of *Spirostachys* is low, while *Grewia* and *Acacia* have high pollen signals (Figure S6). Interestingly, this phase is preceded by high abundances of *Tribulus*, which suggests a transition phase.

Given the changes in species composition and in the grass/woody ratio observed in our study, our results provide empirical evidence to support the hypothesis of Rohde and Hoffman (2012) that transitions from an open woodland into an encroached state in savannas from northern Namibia may occur on a scale of one century and without reversals to a grassy state.

### 4.3 Triggers and drivers

**Figure 5:**
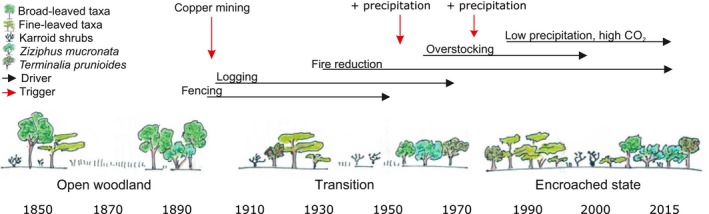
Network of triggers and drivers of savanna vegetation change

It is likely that a complex network of global, regional, and local triggers and drivers including land management (forestry, husbandry/cultivation), precipitation, and atmospheric *p*CO_2_ caused the observed vegetation turnover in the Tsumeb area (Figure 5). However, limited data availability prevents us from doing straightforward multivariate statistical analyses such as decomposition of explained variances to statistically relate driver changes to changes in vegetation (Tian, Herzschuh, Mischke, & Schlütz, 2014); instead, only trends can be compared.

#### 4.3.1 Forestry in the context of mining

Our results support previous studies that assign the emergence of shrubland in northern Namibia to forest management (Strohbach, 2001). In particular, the expansion of thickets in the Tsumeb region, as indicated in forest reports from the 1950s, has been assigned to selective and broad‐scale logging in the course of mining in the early 20th century (Erkkilä, 2001; Lau & Reiner, 1993). *Combretum, Spirostachys africana,* and *C. mopane* were cut for props and fuel for the Tsumeb mine (Erkkilä & Siiskonen, 1992), and this is reflected in the decline of these taxa in the Otjikoto pollen record. After logging, it is likely that dense thickets of *S. africana* formed because of its ability to form stump shoots and root suckers (Gandiwa, Gandiwa, & Mxoza, 2012; Moyo et al., 1993). The *Spirostachys/*Hippomaneae peaks in the pollen and *sed*aDNA records may indicate resprouting events during the Depression and the Second World War times, in which the mine production came to standstill (Lau & Reiner, 1993). In contrast, other local taxa became reduced because of their low growth rates (e.g., *C. mopane*, Cunningham, 1996) or their weak coppicing ability (e.g., *Combretum,* Strohbach, 2001). In order to supply the mines, *Eucalyptus* was planted in the Tsumeb region from the beginning of the 20th century (Dinter, 1909, 1918) and from 1949 at Lake Otjikoto (Lau & Reiner, 1993), which is reflected in our proxy records (Myrtaceae/Myrtoideae). Later on, gradual environmental changes, such as changes in the tree species composition and the reduction of soil moisture as a consequence of deforestation (Moyo et al., 1993), may have favored the spread of *Vachellia, Senegalia,* and *T. prunioides*, which are better adapted to drier conditions.

#### 4.3.2 Husbandry

Land‐use records from this study and census information (FAO, 2018b; Lange, Barners, & Motinga, 1998) indicate that the intensity of husbandry increased throughout the 20th century, which likely affected grass species composition and productivity.

The signal of *Sordaria,* an obligate coprophilous fungus spore associated with dung deposits of wild/domestic herbivores (Dietre, Gauthier, & Gillet, 2012; van Geel & Aptroot, 2006; van Geel et al., 2011), suggests the continuous presence of herbivores in the vicinity of the lake. The high values of *Sordaria* in the first half of the 20th century could be the result of herding of cattle at watering points and on productive pastures close to the lake, particularly during drought episodes (as suggested by the fungal peak at about 1946 [Figures 2 and 4]). Surprisingly, the signal of *Sordaria* decreases in the second half of the 20th century although grazing intensity was likely highest during this period—census information on cattle in Namibia indicates an increase from ~700k animals before 1930 to ~2500k after 1950 (FAO, 2018b; Lange et al., 1998).

The decrease in the *Sordaria* signal could be related to a decline of transhumance pastoralism (as similarly observed by Gelorini, Ssemmanda, & Verschuren[, 2012] at lakes in Uganda), which is associated with changes in land tenure and management in the region. During the first half of the 20th century, most of the Otjikoto region became freehold land (Mendelsohn, Jarvis, Roberts, & Robertson, 2009), which resulted in the construction of fences, concentration of livestock, and eventually the restriction of traditional transhumance in the surroundings of the lake. From the 1960s, movement of cattle was further restricted because of the outbreak of foot‐and‐mouth disease (Schneider, 2012). The increase of cattle in the mid‐20th century likely originated from the intensification of farming from the 1950s (Lau & Reiner, 1993). Additionally, the introduction of subsidies for feed and water‐hole exploration in response to the prolonged drought in the early 1960s (Figure 4) allowed ranchers to increase their stocks in years with good rain, and to maintain them in the drought years (Schneider, 2012; Sweet, 1998).

As reflected in the *sed*aDNA record, these practices led to the decrease of highly palatable grasses such as *Schmidtia* (likely *S. papophoroides*), *B. deflexa,* and *F. africana*. They were, if at all, replaced by less palatable grasses such as *H. dissoluta* and *H. contortus* which are characteristic of overgrazed areas (Kgosikoma, Mojeremane, & Harvie, 2015). High grazing pressure on pastures during the mid‐20th century is also indicated by above‐average signals of nonpalatable taxa such as *Geigeria* (Strohbach & Kutuahuripa, 2014) and *Acanthospermum* (Tolsma, Ernst, & Verwey, 1987), as well as by trampling‐resistant taxa such as *Alternanthera* and *Tribulus/*Tribuloideae (Tabares et al., 2018). High grazing pressure alongside poor grazing resources would also explain the cultivation of *Medicago* to complement livestock fodder from the 1990s around Lake Otjikoto as indicated in the *sed*aDNA record (Figure 3).

The persistent high grazing pressure on pasture with already low grass density may have lowered the seed production of grasses to such an extent that potential niches could not be (re)invaded (O’Connor & Pickett, 1992; Tessema, de Boer, & Prins, 2016). This might explain why our *sed*aDNA record lacks *F. africana* and *Schmidtia* (likely *S. papophoroides*) toward the present day. According to the state‐transition models, the lack of both species may also reflect the vegetation's response to overgrazing when disturbance passed a threshold.

#### 4.3.3 Fire management

Our results show that there were changes in fire management related to husbandry and cultivation near Lake Otjikoto, which further altered the resilience of *Combretum* woodlands and supported shrub establishment in the long‐term.

High macrocharcoal flux and high *Gelasinospora* values (a fungus growing preferentially on burned wood [Revelles & van Geel, 2016]) in sediments of the early 20th century point to local forest fires within a radius of about 5 km from the lake (Clark, 1988; Duffin, Gillson, & Willis, 2008). The emergence of cleared areas is reflected in the *sed*aDNA signals of pioneer grasses such as *B. deflexa* and *Tragus*. During the early 20th century, these areas were likely burned prior to crop cultivation as indicated by records of *Sorghum* (Lau & Reiner, 1993). Landscape opening is also reflected by the aeolian input of well‐sorted coarse silt (rEM3), which was probably blown in from burned fields (Ravi et al., 2009).

During the mid‐20th century, the coincidence of lower charcoal flux and a decrease in *Sorghum sed*aDNA suggest that fire was used to manage livestock grazing by fostering the establishment of palatable grasses (Lau & Reiner, 1993; Sweet, 1998). This would explain the rise in *sed*aDNA signals of *Cenchrus* (likely *C. ciliaris* (Gilo & Kelkay, 2017)) and *Panicum* (likely *P. maximum*) in the second half of the 20th century. Both these highly palatable perennial grasses are common in the *T. prunioides* shrubland (Strohbach, 2014) and are usually planted for hay and pasture (van Oudtshoorn, 2016; Sweet & Burke, 2006). The expansion of monoculture farming in the Tsumeb region, particularly from the 1980s (Lau & Reiner, 1993), is also suggested by decreases in charcoal flux and *Gelasinospora* signals (Figures 2 and 3) toward zone 3, which in turn suggest the suppression of wildfire and/or the reduction of controlled fires to conserve pastures for livestock (Joubert, Smit, & Hoffman, 2012).

An initially reduced grass cover in combination with artificial fire suppression may have affected the grass/shrub competition by reducing mortality of woody seedlings and saplings (Case & Staver, 2017; Joubert et al., 2012). The remaining grass tussocks may have improved the survival of seedlings of encroaching Fabaceae taxa such as *Crotalaria* and *Prosopis*, as the tussocks provide soil moisture and protection against heat stress (De Dios, Weltzin, Sun, Huxman, & Williams, 2014; Wagner, Richter, Joubert, & Fischer, 2018), triggering a vegetation change into an encroached state.

#### 4.3.4 Precipitation and atmospheric CO_2_ changes

Comparing the pollen and *sed*aDNA records of *Vachellia* and *Senegalia* with the mean annual precipitation curve (Figure 4), it seems plausible that drought years from 1980 onwards contributed to the spread of these drought‐tolerant taxa. Previous periods with consecutive rainy summers (particularly 1976–1979 and 1954–1956) and reduced grass cover also favored the germination and establishment of their seeds and saplings. These results are in line with several studies, which identified a positive relationship between rainfall, seed production, and seedling survival of encroacher species (Joubert et al., 2008; Joubert, Smit, & Hoffman, 2013; Wagner et al., 2018).

Our records show a die‐back of *Senegalia* in the 21st century. This may be related to persistent drought stress, as reported from Botswana in 2013 (Joubert et al., 2013). Persistent drought can also inhibit the recovery of *Cenchrus ciliaris, C. apiculatum,* and *D. cinerea* (O’Connor, 1998), which explains the low *sed*aDNA signals of related taxa from the mid‐1990s (i.e., zone 3).

Interestingly, the *Vachellia* pollen and *sed*aDNA signals in zone 3 are high when compared with *Senegalia*, which probably reflects differences in drought sensitivity. As observed by Joubert et al. (2013), *S. mellifera* has a lower tolerance for prolonged periods of moisture stress than *Vachellia* species such as *V. reficiens, V. erubescens,* and *V. erioloba*. According to the authors, *S. mellifera* mostly relies on an extensive shallow‐root system, while *V. reficiens* and *V. erioloba* rely on a deeper tap‐root system, which enables water uptake from deep soil layers.

The observed depletion of δD values of plant‐derived *n*‐alkanes since around 1992 (Figure 4) may also reflect the water uptake of deep‐rooted trees from deep soil layers. In arid and semiarid environments, soil water has higher δD values than precipitation because of strong evaporation (Da Silveira Lobo Sternberg, 1988). Consequently, water from soil surface layers is more enriched in D compared to soil water from deep layers (De Deurwaerder et al., 2018; Ehleringer & Dawson, 1992). Therefore, under dry conditions, xylem water from grasses is expected to have higher δD values compared to trees with a deep root system. Field studies performed in Namibian savannas show that xylem water from deep‐rooted species such as *V. erioloba*, *V. hebeclada,* and *Prosopis juliflora* comes from D‐depleted deep soil layers (Kanyama, 2017). Our pollen and *sed*aDNA results indicate an increase of *Vachellia* and *Prosopis* in zone 3. However, it is unclear to what extent the isotope composition of xylem water is reflected by leaf lipids. While greenhouse experiments found a correlation between leaf wax and soil water δD composition (Hou, D’Andrea, & Huang, 2008), field studies show that the leaf wax δD signature depends mostly on evaporative enrichment of leaf water during photosynthesis (Feakins & Sessions, 2010).

Considering the influence of photosynthesis on δD values, depletion in zone 3, particularly since the beginning of the 21st century, may also reflect an increase in the water‐use efficiency of woody vegetation, which in turn is enhanced through, for example, reduced stomatal conductance and stomata density due to an increasing atmospheric *p*CO_2_ concentration (Figure 4). Studies conducted in tropical environments have shown that trees increase their water‐use efficiency at higher CO_2_ concentrations, despite high transpiration rates (Brienen, Wanek, & Hietz, 2011; van der Sleen et al., 2014). In contrast, trees in arid and semiarid environments, and under higher CO_2_ concentrations, reduce their stomata opening while carbon assimilation increases (Evans, Schortemeyer, McFarlane, & Atkin, 2000; Krishnamurthy & Machavaram, 2000). This improved ratio of carbon assimilation‐to‐conductance results in an increase of transpiration efficiency (Evans et al., 2000), which in turn leads to ^1^H enrichment in leaves and thus a decrease in δD values. Such higher atmospheric CO_2_ concentrations may have favored the propagation of *Vachellia* and *Senegalia* toward the present day, because Fabaceae species are expected to benefit from such conditions in nutrient‐limited savanna environments (Wagner et al., 2018; Ward, Hoffman, & Collocott, 2014). As observed by Evans et al. (2000), *Acacia* species increase their nitrogen content per unit foliage area when growing under a higher CO_2_ concentration. Consequently, the photosynthetic capacity of leaves increases (Evans, 1989), which can then improve the competitiveness of *Acacia* species against grasses.

The interpretation of the leaf wax δD is complicated further as the signature of the water source of plants may have changed as well. The high δD values around 1916 (i.e., in the transition between zones 1 and 2) probably reflect an enrichment of source water. Such an interpretation is supported by the high concentration of inorganic carbon (TIC) and an increase of *Ficus* pollen that inhabits the walls of the sinkhole. A low lake level may result from low rainfall between 1916 and 1922 (Figure 4). The decrease of δD values from the mid‐1990s (i.e., at the transition to zone 3) despite high TIC and *Ficus* pollen signals and low precipitation, however, contradicts the interpretation of the leaf wax δD as primarily representing a precipitation signal (Niedermeyer et al., 2016; Sachse et al., 2012) but rather supports our interpretation of changes in the plant water source.

Our results suggest that environmental changes derived from management (i.e., reduced soil moisture, reduced grass cover, changes in species composition and competitiveness, reduced fire intensity) may have affected the resilience of *Combretum* woodlands, making them more susceptible to change into an encroached state by stochastic events such as consecutive years of precipitation and drought, and by high *p*CO_2_ concentrations. These results are in line with state‐transition models, which predict gradual changes in environmental conditions to alter the resilience of stable states, thus enhancing the likelihood of a state change (Scheffer et al., 2001).

### 4.4 State‐stabilizing feedbacks

Comparing our multiproxy record with historical climate and land‐use data allows us to infer the feedback mechanisms that support the stable states observed in our study. This allows us to interpret the observed vegetation changes in the context of state‐transition models (Hirota, Holmgren, Van Nes, & Scheffer, 2011; Scheffer et al., 2001).

#### 4.4.1 Open *Combretum* woodland

Transhumance and intense fires, as reflected by our spore and charcoal records, may have stabilized an open woodland phase. A high grass cover, as observed in phase 1, may produce enough fuel for intense fires, which increase seedling and sapling mortality (Beckett & Bond, 2019; Case & Staver, 2017; Hirota et al., 2011; Staver, Archibald, & Levin, 2011). Since tree seedlings are not as fire‐resistant as adult trees and some of them are very palatable (e.g., *Dichrostachys*), a combination of fire and browsing may also have prevented tree seedlings from being recruited to mature age classes (Gillson, 2004; Joubert et al., 2012; Scheffer et al., 2001).

In addition, wetter conditions may have favored competitiveness of *Combretum* and *Spirostachys* against drought‐tolerant taxa. An early *Combretum*‐*Spirostachys* woodland phase at Otjikoto was linked to wetter climatic conditions (Scott et al., 1991). Although vegetation models predict a woody state in savannas with increased precipitation (Hirota et al., 2011), our results allow us to infer that under such climatic conditions open *Combretum* woodland is possible, but also *Terminalia* shrubland, as the latter profits from wet years followed by persistent drought.

#### 4.4.2 Terminalia shrubland

Several feedback mechanisms may stabilize the encroached state and inhibit the reverse transition toward an open woodland state:

First, open patches in degraded pastures may have supported the formation of trampling‐resistant soil crusts (Dougill & Thomas, 2004) containing nitrogen‐fixing cyanobacteria. These soil crusts, in turn, may have favored the establishment of nitrogen‐fixing shrubby Fabaceae, which eventually inhibit grass growth under their canopy (Maron & Connors, 1996). Such a feedback mechanism would explain the decrease in the C/N ratio (Figure 4) in the uppermost sedimentary record due to the allochthonous input of nitrogen‐enriched soil materials (Talbot, 2005) in Lake Otjikoto. The latter is also supported by the predominance of fine‐sized sediments in zone 3 (rEM1, rEM2), which may also depress the C/N values by uptake of inorganic N, since higher proportions of clay can absorb ammonia well (Meyers, 1997).

Second, the reduction of grass cover toward the present day in Tsumeb coincides with soil erosion, as suggested by *Glomus*, a spore of an arbuscular mycorrhizal fungus associated with eroded soils (Revelles & van Geel, 2016; van Geel et al., 2011) (Figure 2). On bare soil patches with soil crusts, run‐off increases and water infiltration decreases, which further reduces the establishment of grass seedlings (Strohbach, 2001). In addition, climate‐ and/or human‐induced damage to soil crusts can lead to soil losses (as suggested by rEM4 in zone 3), which in turn promote rill formation thus reducing the surface run‐off and limiting the run‐on input to vegetation (i.e., limiting the formation of temporary ponds and soils with water tables (Rodríguez‐Caballero et al., 2012; Valentin, 1993)). Our study supports previous studies that find soil erosion in the course of bush encroachment since the second half of the 20th century (De Klerk, 2004; Strohbach, 2001).

Third, the practice of bush removal (indicated for Lake Otjikoto by a slight decrease of shrub pollen in the uppermost core section and observed in the locality of Otjiguinas Farm during fieldwork) enables the establishment of fast‐growing species such as *H. dissoluta* (recorded in the uppermost section). This perennial grass spreads across disturbed fields forming dense tall swards, which inhibit the growth of other grasses (Cech, Edwards, & Olde Venterink, 2010; Jordaan, 2017).

Fourth, once established, woody vegetation with a mixed rooting system, such as *S. mellifera* and *Prosopis* sp., can suppress grass production (Smit, 2003), as they can effectively take up soil moisture from the upper soil layer after rain events with their lateral roots and survive dry phases due to water uptake by deep roots (Ansley, Boutton, & Jacoby, 2014; Brown & Archer, 1999). A reduced grass cover in turn reduces the intensity of fire, resulting in the self‐propagation of a woody state (Higgins & Scheiter, 2012; Hirota et al., 2011; Scheffer et al., 2001).

Fifth, climate change (i.e., higher atmospheric *p*CO_2_ concentration and changes in precipitation variability) may induce physiological adaptations (as suggested by our δD record) enhancing the competitiveness of trees over grasses. Although persistent drought constrains the CO_2_‐related photosynthetic advantages for savanna trees (Nackley et al., 2018), the assimilation rates are still higher for C_3_ plants compared to C_4_ grasses under such conditions (Bellasio, Quirk, & Beerling, 2018). This is because C_4_ grasses under water restriction experience metabolic inhibitions that have a greater effect on leaf assimilation compared to the limited stomatal conductance of savanna trees (Bellasio et al., 2018). Furthermore, under restored watering conditions, photosynthetic rates recover faster when inhibited by stomatal than by metabolic factors, which in turn affects the competitiveness of C_4_ grasses under optimal conditions (Bellasio et al., 2018). Such a feedback mechanism implies that at higher atmospheric *p*CO_2_ concentration, an increase in the frequency of droughts and heavy rains may favor the expansion of C_3_ vegetation (Zhang et al., 2019).

These feedback mechanisms explain the ongoing encroachment processes observed at our study site up to the present, even though management intensity may have adjusted to the local carrying capacity (e.g., decline of cattle numbers from the 1970s (Lange et al., 1998) and a levelling off of cattle populations since 2000) or decreased (e.g., regulation of timber production since 1968 (Erkkilä & Siiskonen, 1992)). The current vegetation may thus represent a management legacy from the first half of the 20th century, as well as the impact of climate change.

## 5 CONCLUSIONS

We have shown that the expansion of shrubs is a multidecadal trend characterized by the turnover from stable open woodland to a stable encroached state. Our results indicate that such a state change may occur on a scale of a century and without short‐term reversions to a grassy state, but with an unstable transition phase, which covers about 80 years and is characterized by a steady turnover of taxa, some of which are restricted to the transition phase.

We demonstrate that the current grass/woody ratio is exceptional for the last 170 years and that such a process is related to gradual changes in soil moisture, erodibility, and species composition and competitiveness. Our inferences largely support prior theoretical considerations about shrub encroachment dynamics in southern African savannas that were mostly based on space‐for‐time approaches or studies with low taxonomic and temporal resolution.

Our study shows, for the first time, the potential of *seda*DNA analysis to track vegetation changes in savanna environments, as well as to infer related local and global triggers and drivers. In particular, detailed taxonomical information enabled us to confirm and complement our pollen findings, and thus to reconstruct the turnover from broad‐leaved to fine‐leaved tree taxa and *Terminalia.*

Taking into account an earlier pollen record of Lake Otjikoto (Scott et al., 1991), we suggest that shrub encroachment is not a new phenomenon and that it possibly occurred in the past in the Tsumeb region, as suggested by changes in taxonomic composition and the grass/woody ratio observed in the previous record. In this sense, the encroached state observed in our study can be interpreted as a successive phase in a context of state transitions and is in line with previous studies that define encroachment as an alternative stable state (Gil‐Romera et al., 2010).

Comparing our vegetation proxies to environmental proxies and historical data, we inferred the impact of logging, as well as physiological adaptations related to changes in precipitation regime and high atmospheric *p*CO_2_ concentrations. Furthermore, detailed information of grass taxonomical composition (which is restricted in the pollen record) made it possible to infer changes in land use, such as crop cultivation, selective grazing, and overgrazing. Combined with our macrocharcoal record, we deduce changes in fire management associated with the intensification of farming.

Our study helps fill a knowledge gap about the feedback mechanisms supporting shrub encroachment. Such mechanisms reflect the interplay between historical land use and climate change (Perring et al., 2018), enabling us to confirm the far‐reaching effects of management legacies on vegetation in savanna ecosystems.

Overall, this study highlights the suitability of multiple lake sedimentary proxies (particularly *sed*aDNA) to resolve complex compositional change and related environmental conditions in semiarid savannas on at least decadal and centennial timescales.

## CONFLICT OF INTEREST

None declared.

## AUTHOR'S CONTRIBUTIONS

XT and UH conceived the ideas and designed methodology; XT and LD performed fossil pollen and macrocharcoal analyses; HZ and XT performed *seda*DNA analyses; XT and ED performed grain‐size and end‐member modeling analysis; LB, AV, HW, and XT performed biogeochemical and sedimentological analyses. Under supervision of UH, XT wrote a first version of the manuscript that all coauthors commented on. All authors gave final approval for publication.

## Data Availability

The data that support the findings of this study are openly available in PANGEA repository: Tabares, X., Zimmermann, H., Dietze, E., Ratzmann, G., Belz, L., Vieth‐Hillebrand, A., Dupont, L., Wilkes, H., Mapani, B., Herzschuh, U. (2019): Palynology, sedimentary ancient DNA, biomarkers, macrocharcoal, and sedimentological data from Lake Otjikoto, Namibia. PANGAEA, https://doi.org/10.1594/PANGAEA.902988.
