# Supplementary material for: Vegetation state changes in the course of shrub encroachment in an African savanna since about 1850 CE and their potential drivers
Source: Ecol Evol. 2019 Dec 30;10(2):962–79. doi: 10.1002/ece3.5955 (PMC6988543; doi:10.1002/ece3.5955)
Supplement: Supplementary file 1 [file ECE3-10-962-s001.docx]

**Supplement 1. Stable isotope analyses - methodological details.**

**A. Compound specific carbon (δ^13^C) and deuterium (δD) isotope analysis**

The carbon and deuterium isotopic compositions of saturated hydrocarbons were measured by GC-IRMS, which is composed of a GC unit (7890N, Agilent Technology, USA) connected to a GC-Isolink coupled via open split to a Delta V Plus mass spectrometer (ThermoFisher Scientific, Germany). For carbon isotope analysis, the organic substances of the GC effluent stream were oxidised to CO_2_ in the combustion furnace held at 940°C on a CuO/Ni/Pt catalyst, whereas the organic compounds were pyrolysed at 1450°C to obtain ^2^H for deuterium isotope analysis. We injected 3 µl of saturated fraction to the programmable temperature vaporisation inlet (PTV, Agilent Technology, USA) with a septumless head, working in split/splitless mode. The injector was held at a split ratio of 1:2 and a constant temperature of 300°C. The saturated fractions were separated on a fused silica capillary column (HP Ultra 1, 50 m x 0.2 mm ID, 0.33 µm FT, Agilent Technology, Germany). The temperature programme started at 80°C, held for 2 minutes and was then increased at a rate of 5°C min^-1^ to 325°C, held for 15 minutes. Helium, set to a flow rate of 1.3 ml min^-1^, was used as a carrier gas. All saturated hydrocarbon fractions were measured in triplicate for carbon and deuterium isotope analysis and the standard deviation was ≤0.5‰ for most of the compounds and samples for δ^13^C and ≤3‰ for δD. The quality of the isotope measurements was checked regularly by measuring different *n*-alkane standards with known isotopic composition (provided by Campro Scientific, Germany and Arndt Schimmelmann, Indiana University, USA).

**B. Bulk carbon (δ^13^Corg) isotope analysis**

For analyses of carbon isotope ratios of sedimentary organic matter (δ^13^Corg), samples were decarbonized with HCL (7%) at 80°C. Samples were weighed into tin capsules and measured using an elemental analyser (1108 from Carlo Erba) coupled to a MAT 252 isotope ratio mass spectrometer from Thermo Fisher Scientific. All samples were analysed in duplicate. The calibration was performed using a certified isotope standard (IAEA CH-6 with a δ^13^C value of -10.45‰). Instrument performance was checked using a sediment reference sample with a δ^13^Corg value of -26.07‰ (IVA). The reproducibility for replicate analyses was 0.5‰.

**Supplement 2. Grain size end-member modelling**


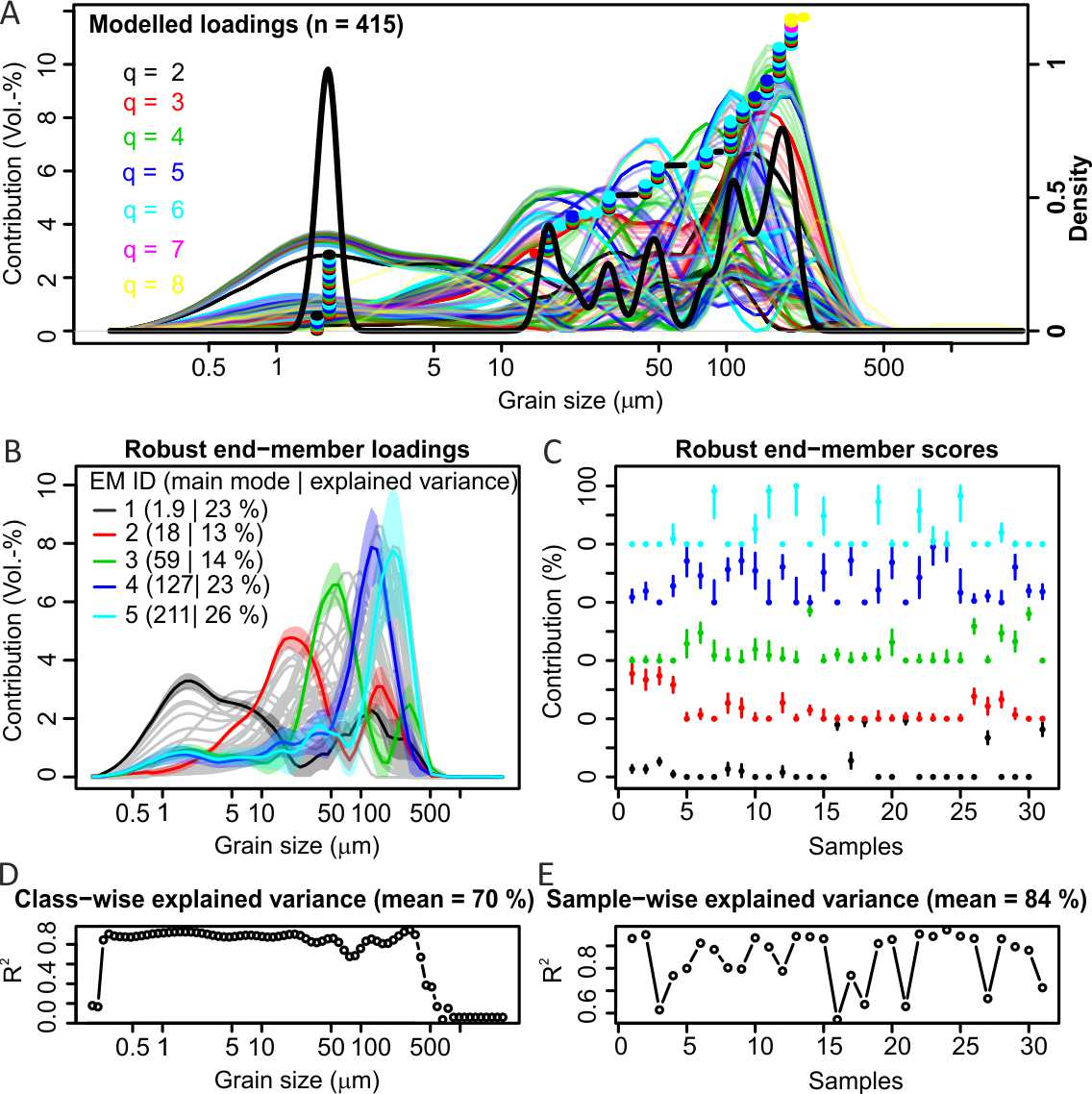


Figure S2. Grain size end-member modelling of 15OJ10 sediment core. A) All modelled end-member loadings that are similarly likely for different numbers of end-members (coloured) for a model parameter space between a minimum and maximum number of end-members and weight transformation value (after Dietze and Dietze, in press). Dots mark respective main modes. Thick black line is a kernel density estimate that shows robust end-members, i.e. high density at loading with similar main modes. B) Robust end-member loadings, class-wise end-member contributions, defined after the five highest mode positions in A. C) Robust end-member scores (mean and one standard deviation) are the relative contributions of an end-member to each sample. D) and E) show the class- and sample-wise explained variance of the mean robust end-member model.

**Supplement 3. Radiometric chronology of the Lake Otjikoto**

A.


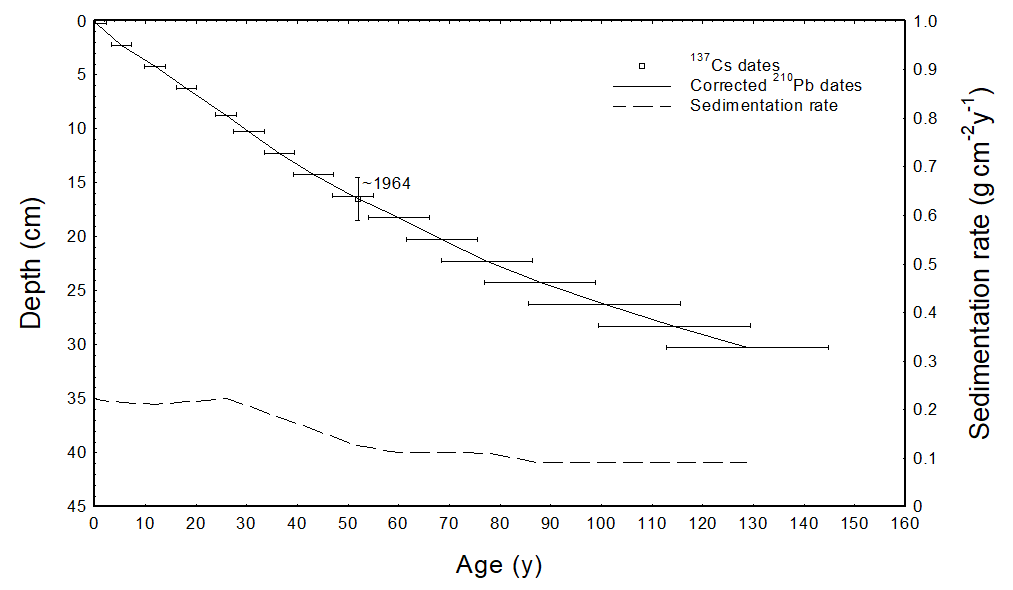


B.


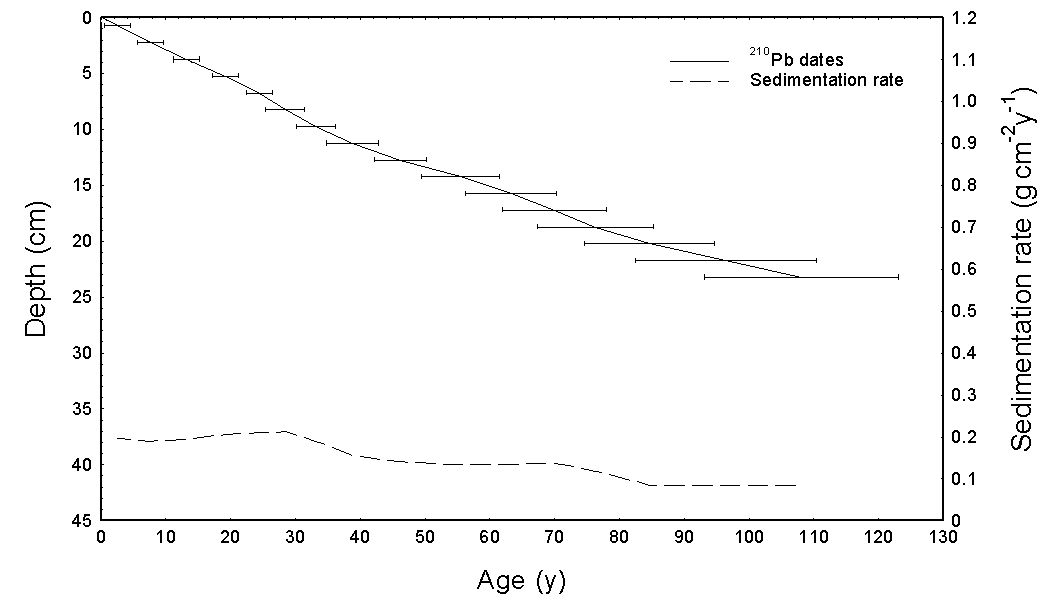


Figure S3. Radiometric chronology of Lake Otjikoto showing the corrected CRS model ^210^Pb dates and sedimentation rates for A) core 15OJ10 and B) core 15OJ06.

**Supplement 4. Pollen and spores**

| **Pollen and spores** | |  |  |
| --- | --- | --- | --- |
| *Abutilon* | *Crotalaria* | Malvaceae | Rubiaceae |
| *Acalypha* | *Croton* | *Medicago* | *Ruellia* |
| Acanthaceae | Cucurbitaceae | *Melhania* | Scrophulariaceae |
| *Adenium* | Cyperaceae | Meliaceae | *Senecio* |
| *Afzelia* | *Dichrostachys* | *Monsonia* | *Senegalia* |
| Aizoaceae | *Diptychandra* | *Montinia* | *Sida* |
| *Alchornea* | *Dodonea* | Mopane | *Solanum* |
| *Aloe* | *Ecbolium* | Myrtaceae | *Spirostachys* |
| *Alternanthera* | *Ehretia* | *Nuxia* type | *Talinum* |
| Anacardiaceae | *Euclea* | Nyctagynaceae | *Tapinanthus* |
| Apiaceae | *Euphorbia* type | *Ocimum* | *Tarchonanthus* |
| Aquatics | Euphorbiaceae undeff | *Opilia* | *Tephrosia* |
| *Arctotis* | *Ficus* | *Opuntia* | *Tragia* |
| *Bauhinia* | *Gazania* type | Orobanchaceae | *Triaspis* |
| Bignoniaceae | *Grewia* | *Parkinsonia* | *Tribulus* |
| *Blepharis* | *Grubbia* | Pedaliaceae | *Vachellia* |
| Brassicaceae | *Gymnosporia* | *Pentzia* | Verbenacea |
| *Burkea* | *Heeria* | *Petalidium* | *Waltheria* |
| Capparaceae | *Heliotropium* | *Philenoptera* | *Xanthium* type |
| *Chamaecrista* | *Hyphaene* | *Phyllanthus* | *Ximenia* |
| Chen./Amara. | *Hypoestes* | Poaceae | *Ziziphus* |
| Cichorioideae | *Justicia* | *Podocarpus* | *Acrodyctis* |
| Combretaceae | *Kalanchoe* | Polygonaceae | *Cercophora* |
| *Commelina* | *Kirkia* | Portulacaceae | *Curvularia* |
| *Commiphora* | *Kohautia* | *Prosopis* | *Gelasinospora* |
| Convolvulaceae | Lamiaceae | Proteaceae | *Glomus* |
| *Cordia* | *Limeum* | *Pterospermum* | *Neurospora* |
| *Corymbium* | *Logania* type | *Rhus* | *Sordaria* |
| Crassulaceae | Lythraceae | *Rhynchosia* | *Spegazzinia* |

Table S4. List of identified pollen and spore taxa.

**Supplement 5. *seda*DNA taxa**

| **sedaDNA taxa** |  |  |  |  |
| --- | --- | --- | --- | --- |
| **Family** |  |  |  |  |
| Acanthaceae | Chenopodiaceae | Lamiaceae | Rhamnaceae | |
| Amaranthaceae | Convolvulaceae | Loranthaceae | Sapindaceae | |
| Anacardiaceae | Cucurbitaceae | Lythraceae | Scrophulariaceae | |
| Apocynaceae | Cupressaceae | Meliaceae | Solanaceae |  |
| Asparagaceae | Ebenaceae | Moraceae | Typhaceae |  |
| Asteraceae | Ehretiaceae | Musaceae | Verbenaceae | |
| Bignoniaceae | Euphorbiaceae | Myrtaceae | Vitaceae |  |
| Brassicaceae | Fabaceae | Opiliaceae |  |  |
| Burseraceae | Heliotropiaceae | Pedaliaceae |  |  |
| Capparaceae | Hyacinthaceae | Ranunculaceae |  |  |
|  |  |  |  |  |
| **Genus** |  |  |  |  |
| *Acalypha* | *Cordia* | *Hibiscus* | *Panicum* | *Solanum* |
| *Aristida* | *Croton* | *Hypoestes* | *Persea* | *Sorghum* |
| *Arthraxon* | *Cyphostemma* | *Lepidium* | *Phaseolus* | *Stuckenia* |
| *Astrantia* | *Dichrostachys* | *Leucaena* | *Phylica* | *Tagetes* |
| *Bougainvillea* | *Duranta* | *Lycopersicon* | *Pisum* | *Terminalia* |
| *Calostephane* | *Ehretia* | *Maprounea* | *Poaceae* | *Themeda* |
| *Capsicum* | *Eriocephalus* | *Medicago* | *Polygonum* | *Thunbergia* |
| *Celastraceae* | *Euphorbia* | *Mentzelia* | *Pyrus* | *Tragus* |
| *Cenchrus* | *Geigeria* | *Mirabilis* | *Quercus* | *Vachellia* |
| *Combretum* | *Gossypium* | *Monsonia* | *Schmidtia* | *Zaleya* |
| *Commelina* | *Grewia* | *Muhlenbergia* | *Senegalia* |  |
| *Corchorus* | *Heliotropium* | *Oxygonum* | *Sida* |  |
|  |  |  |  |  |
| **Species** |  |  |  |  |
| *Abutilon mauritianum* | | *Dodonaea viscosa* | | *Parkinsonia anacantha* |
| *Acanthospermum australe* | | *Euphorbia hypericifoliae* | | *Paspalum notatum* |
| *Aegopodium podagraria* | | *Fingerhuthia africana* | | *Philenoptera cyanescens* |
| *Aloe grandidentata* | | *Garnotia tenella* |  | *Phyllanthus* |
| *Alternanthera pungens* | | *Glyphochloa talbotii* | | *Pithecellobium* sp. DS14533 |
| *Antigonon leptopus* | | *Grubbia rosmarinifolia* | | *Portulaca oleracea* |
| *Aucoumea klaineana* | | *Haematoxylum dinteri* | | *Psidium guajava* |
| *Baratranthus axanthus* | | *Helinus integrifolius* | | *Pterospermum heterophyl.* |
| *Bellida graminea* |  | *Heteropogon contortus* | | *Saccharum spontaneum* |
| *Beta vulgaris* |  | *Hyperthelia dissoluta* | | *Schistocarpaea johnsonii* |
| *Blepharis maderaspatensis* | | *Ipomoea cairica* |  | *Senegalia senegal* |
| *Brachiaria deflexa* | | *Kirkia acuminata* | | *Senna gardneri* |
| *Cassinopsis ilicifolia* | | *Lactuca sativa* |  | *Spermacoce princeae* |
| *Castilleja vadosa* |  | *Leucosphaera bainesii* | | *Stemodiopsis buchananii* |
| *Catophractes alexandri* | | *Malosma laurina* | | *Stilbe rupestris* |
| *Citronella smythii* | | *Mecomischus halimifolius* | | *Tribulus terrestris* |
| *Combretum coccineum* | | *Melhania ovata* |  | *Vachellia hebeclada* |
| *Commicarpus pedunculosus* | | *Meum athamanticum* | | *Veronica anagalloides* |
| *Conocarpus erectus* | | *Montinia caryophyllacea* | | *Vicia faba* |
| *Cucurbita pepo* |  | *Nama demissa* |  | *Waltheria indica* |
| *Cyperus congestus* | | *Neyraudia reynaudiana* | |  |
| *Diptychandra aurantiaca* | | *Pachira quinata* |  |  |
|  |  |  |  |  |
| **Subfamily** | **Subtribe** | **Tribe** |  | **No rank** |
| Apioideae | Justiciinae | Acacieae | Lantaneae | PACMAD clade |
| Asclepiadoideae |  | Andropogoneae | Phyllantheae | |
| Malvoideae |  | Caesalpinieae | Pomaderreae | |
| Mimosoideae |  | Hippomaneae | Ruellieae |  |
| Papilionoideae |  | Indigofereae |  |  |
| Tribuloideae |  | Ipomoeeae |  |  |

Table S5. List of identified *seda*DNA taxa.

**Supplement 6. State-changes at Otjikoto Lake**

**
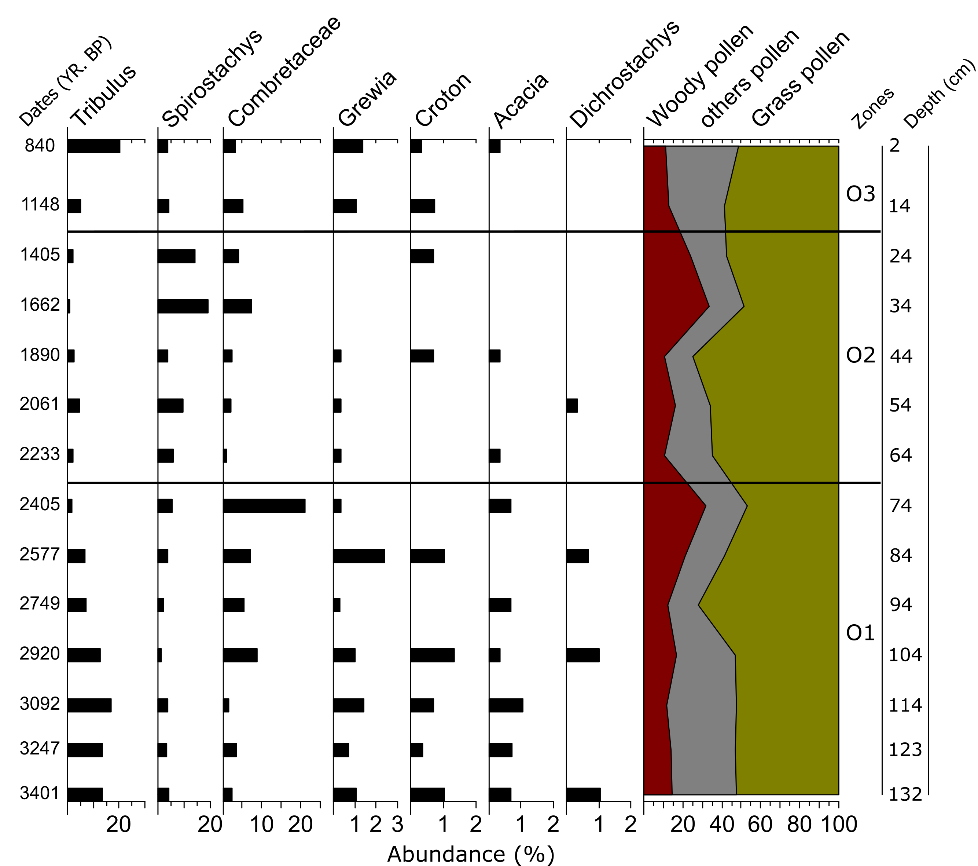
**

Figure S6. Percentage abundance diagram of selected pollen taxa from Lake Otjikoto (Leźine, 2005; Scott et al., 1991). The O1 and O2 phases correspond to woodland phases dominated by Combretaceae and *Spirostachys,* respectively. The woodland phases are separated by periods of open vegetation, as observed in phase O3. Mean abundance percentages of woody, grass, and other pollen taxa are also displayed.
